# Supplementary material for: Polyphenolic Profile of Herniaria hemistemon Aerial Parts Extract and Assessment of Its Anti-Cryptosporidiosis in a Murine Model: In Silico Supported In Vivo Study
Source: Pharmaceutics. 2023 Jan 26;15(2):415. doi: 10.3390/pharmaceutics15020415 (PMC9964224; doi:10.3390/pharmaceutics15020415)
Supplement: Supplementary file 1 [file pharmaceutics-15-00415-s001.zip › pharmaceutics-2118677-supplementary.pdf]

Polyphenolic profile of *Herniaria hemistemon* aerial parts extract and assessment of its anti-cryptosporidiosis in a murine model: *In silico* supported *in vivo* study

Mosad A. Ghareeb [1,*], Mansour Sobeh [2,*], Tarek Aboushousha [3], Marwa Esmat [4], Hala Sh. Mohammed [5] and Eman S. El-Wakil [6]

[1] Medicinal Chemistry Department, Theodor Bilharz Research Institute, Kornaish El-Nile, Warrak El-Hadar, Imbaba (P.O. 30), Giza 12411, Egypt
[2] AgroBioSciences, Mohammed VI Polytechnic University, Lot 660–Hay Moulay Rachid, Ben-Guerir 43150, Morocco
[3] Department of Pathology, Theodor Bilharz Research Institute, Kornaish El-Nile, Warrak El-Hadar, Imbaba (P.O. 30), Giza 12411, Egypt; t.aboushousha@tbri.gov.eg
[4] Department of Medical Parasitology, Faculty of Medicine, Misr University for Science and Technology, 6th October City 12566, Egypt; marwa.esmat@must.edu.eg
[5] Department of Pharmacognosy and Medicinal Plants, Faculty of Pharmacy (Girls), Al-Azhar University, Cairo 11311, Egypt; halashaaban676.el@azhar.edu.eg
[6] Department of Parasitology, Theodor Bilharz Research Institute, Kornaish El-Nile, Warrak El-Hadar, Imbaba (P.O. 30), Giza 12411, Egypt; drfaith@ymail.com
* Correspondence: m.ghareeb@tbri.gov.eg (M.A.G.); mansour.sobeh@um6p.ma (M.S.); Tel.: +20-(02)-010-1234-6834 (M.A.G.); Fax: +20-(02)-35408125 (M.A.G.)

Abstract: *Herniaria hemistemon* J.Gay is widely used in folk medicine to treat hernia. The present study aimed to annotate the phytoconstituents of *H. hemistemon* aerial-part extract and investigate its in vivo anticryptosporidial activity. The chemical characterization was achieved via the LC–ESI–MS/MS technique resulting in the annotation of 37 phytocompounds comprising flavonoids and phenolic acids. Regarding the anticryptosporidial activity, fifty dexamethasone-immunosuppressed mice were separated into five groups: GI, un-infected (normal control); GII, infected but not treated (model); GIII, infected and received NTZ, the reference drug; GIV, infected and received *H. hemistemon* extract (100 mg/kg); and GV, infected and received *H. hemistemon* extract (200 mg/kg). When GIII, GIV, and GV were compared to GII, parasitological analyses displayed highly significant differences in the mean numbers of *Cryptosporidium parvum* oocysts in the stool between the different groups. GV demonstrated the highest efficacy of 79%. Histopathological analyses displayed improvement in the small intestine and liver pathology in the treated groups (GIII, IV, and V) related to the model (GII), with GV showing the highest efficacy. Moreover, the docking-based study tentatively highlighted the potential of benzoic acid derivatives as lactate dehydrogenase inhibitors. The docked compounds showed the same binding interactions as oxamic acid, where they established H-bond interactions with ARG-109, ASN-140, ASP-168, ARG-171, and HIS-195. To sum up, *H. hemistemon* is a promising natural therapeutic agent for cryptosporidiosis.

Keywords: *Herniaria hemistemon* J.Gay; nitazoxanide; anticryptosporidiosis; polyphenols; LC–ESI–MS/MS; lactate dehydrogenase inhibitors

1. Docking-based virtual screening

1.1. Ligand Structure Generation

OpenBabel v.3.1.1 [1] was used to convert the structures' SMILE codes to three-dimensional configurations that were subsequently subjected to a minimization of energy using the steepest descent technique with the same software. The minimization was performed by the force field MMFF94. Using AutoDockTools v.4.2, all torsions of the selected structures were assigned and their Gasteiger charges were provided for all studied atoms in structures [2].

1.2. Protein Structure Preparation

For docking screening, the crystal structure of lactate dehydrogenase from *Cryptosporidium parvum* (PDB code: 4ND1) [3] was used. PDBfixer [4] was used to edit the downloaded structure, adding missing residues and atoms, and removing co-crystalized H_2O and heteroatoms. Through AutoDock Tools v.4.2, polar hydrogen and Gasteiger charges were subsequently made available for both proteins.

1.3. Structural Docking

The docking process was carried out using the PyRx platform's built-in AutoDock Vina software [4,5]. According to the co-crystalized ligands of both enzymes, the docking search grid boxes were determined to perfectly enclose them with a 20 Å^3 total size. The grid box's coordinates were set to be x = 23.5; y = 38.45; z = 64.84. The level of exhaustion was held at 24. Pymol software was used to evaluate and display docking poses. Exhaustiveness was set to 24. Ten poses were generated for each docking experiment. Docking poses were analyzed and visualized using Pymol software [6]. The docking protocol was validated by re-docking the co-crystalized ligand (i.e. oxamic acid) into

the active site of the enzyme. The resulting top-scoring pose of the co-crystalized ligand was in good alignment with the co-crystalized one with slight deviations (RMSDs = 0.74).

References

1. O'Boyle NM, Banck M, James CA, Morley C, Vandermeersch T, Hutchison GR. Open Babel: An open chemical toolbox. J. Cheminform. 2011; 3: 33. https://doi.org/10.1186/1758-2946-3-33.
2. Morris GM, Huey R, Lindstrom W, Sanner MF, Belew RK, Goodsell DS, Olson AJ. AutoDock4 and AutoDockTools4: Automated docking with selective receptor flexibility. J. Comput. Chem. 2009; 30: 2785-2791. https://doi.org/10.1002/jcc.21256.
3. Cook WJ, Senkovich O, Hernandez A, Speed H, Chattopadhyay D. Biochemical and structural characterization of *Cryptosporidium parvum* lactate dehydrogenase. International Journal of Biological Macromolecules. 2015; 74: 608-619. https://doi.org/10.1016/j.ijbiomac.2014.12.019.
4. Eastman P, Friedrichs MS, Chodera JD, Radmer RJ, Bruns CM, Ku JP, Beauchamp KA, Lane TJ, Wang L-P, Shukla D, Tye T, Houston M, Stich T, Klein C, Shirts MR, Pande VS. OpenMM 4: A Reusable, Extensible, Hardware Independent Library for High Performance Molecular Simulation. J. Chem. Theory Comput. 2013; 9: 461-469. https://doi.org/10.1021/ct300857j.
5. Dallakyan S, Olson AJ. In Chemical Biology (Eds: J. E. Hempel, C. H. Williams, C. C. Hong), Springer New York, NY, 2015, pp. 243-250. https://doi.org/10.1007/978-1-4939-2269-7_19.
6. Seeliger D, de Groot BL. Ligand docking and binding site analysis with PyMOL and Autodock/Vina. J. Comput. Aided Mol. Des. 2010; 24: 417-422. https://doi.org/10.1007/s10822-010-9352-6.
